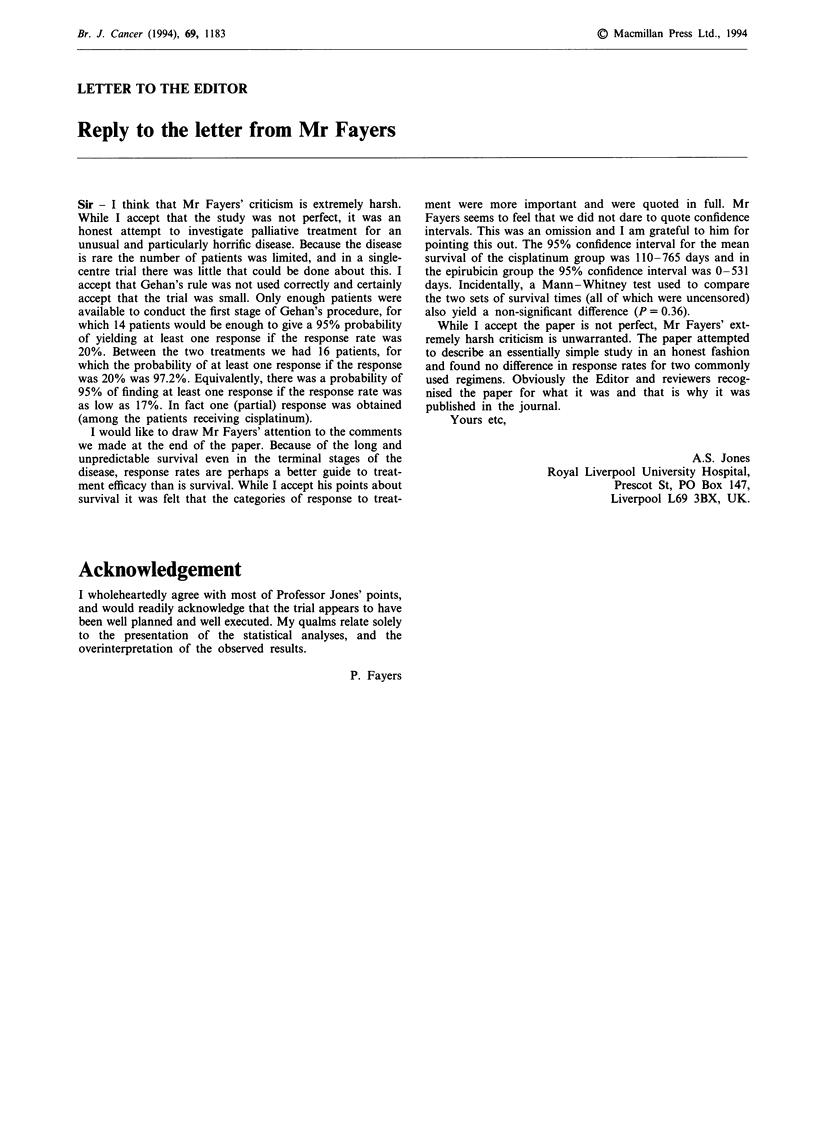# Reply to the letter from Mr Fayers

**Published:** 1994-06

**Authors:** A.S. Jones


 © Macmillan Press Ltd., 1994

LETTER TO THE EDITOR

### Reply to the letter from Mr Fayers

**Sir** – I think that Mr Fayers' criticism is extremely harsh. While I accept that the study was not perfect, it was an honest attempt to investigate palliative treatment for an unusual and particularly horrific disease. Because the disease is rare the number of patients was limited, and in a single-centre trial there was little that could be done about this. I accept that Gehan's rule was not used correctly and certainly accept that the trial was small. Only enough patients were available to conduct the first stage of Gehan's procedure, for which 14 patients would be enough to give a 95% probability of yielding at least one response if the response rate was 20%. Between the two treatments we had 16 patients, for which the probability of at least one response if the response was 20% was 97.2%. Equivalently, there was a probability of 95% of finding at least one response if the response rate was as low as 17%. In fact one (partial) response was obtained (among the patients receiving cisplatinum).

I would like to draw Mr Fayers' attention to the comments we made at the end of the paper. Because of the long and unpredictable survival even in the terminal stages of the disease, response rates are perhaps a better guide to treatment efficacy than is survival. While I accept his points about survival it was felt that the categories of response to treatment were more important and were quoted in full. Mr Fayers seems to feel that we did not dare to quote confidence intervals. This was an omission and I am grateful to him for pointing this out. The 95% confidence interval for the mean survival of the cisplatinum group was 110–765 days and in the epirubicin group the 95% confidence interval was 0–531 days. Incidentally, a Mann–Whitney test used to compare the two sets of survival times (all of which were uncensored) also yield a non-significant difference ($P = 0.36$).

While I accept the paper is not perfect, Mr Fayers' extremely harsh criticism is unwarranted. The paper attempted to describe an essentially simple study in an honest fashion and found no difference in response rates for two commonly used regimens. Obviously the Editor and reviewers recognised the paper for what it was and that is why it was published in the journal.

Yours etc,

A.S. Jones
Royal Liverpool University Hospital,
Prescot St, PO Box 147,
Liverpool L69 3BX, UK.

### Acknowledgement

I wholeheartedly agree with most of Professor Jones' points, and would readily acknowledge that the trial appears to have been well planned and well executed. My qualms relate solely to the presentation of the statistical analyses, and the overinterpretation of the observed results.

P. Fayers